# Disease-Specific Alteration of Cardiac Lymphatics: A Review from Animal Disease Models to Clinics

**DOI:** 10.3390/ijms251910656

**Published:** 2024-10-03

**Authors:** Yuuki Shimizu, Haihang Luo, Toyoaki Murohara

**Affiliations:** Department of Cardiology, Nagoya University Graduate School of Medicine, Nagoya 466-8550, Japan

**Keywords:** lymphatic vessel, heart disease, animal model, clinical report, review article

## Abstract

For many years, the significance of cardiac lymphatic vessels was largely overlooked in clinical practice, with little consideration given to their role in the pathophysiology or treatment of cardiac diseases. However, recent research has brought renewed attention to these vessels, progressively illuminating their function and importance within the realm of cardiovascular science. Experimental studies, particularly those utilizing animal models of cardiac disease, have demonstrated a clear relationship between cardiac lymphatic vessels and both the pathogenesis and progression of these conditions. These findings have prompted a growing interest in potential therapeutic applications that specifically target the cardiac lymphatic system. Conversely, while clinical investigations into cardiac lymphatics remain limited, recent studies have begun to explore their identification through specific surface markers, as well as the expression dynamics of lymphangiogenic factors. These studies have increasingly highlighted associations of lymphatic dysfunction with inflammation and fibrosis, both of which negatively impact cardiac function and remodeling across various pathological states. Despite these advances, comprehensive reviews of the current knowledge regarding the cardiac lymphatic vasculature, particularly within specific disease contexts, remain scarce. This review aims to address this gap by providing a detailed synthesis of existing reports, encompassing both animal model research and studies on human clinical specimens, with a special focus on the role of cardiac lymphatic vessels in different disease states.

## 1. Introduction

Lymphatics are blind-ended vessels and unidirectional ducts that commonly run parallel to blood vessels in the body, forming a network system interconnected with the vascular system. They possess a structure akin to that of blood vessels, comprising three layers: an endothelial layer, a smooth muscle layer, and an outer membrane. Lymphatics maintain tissue fluid homeostasis by absorbing leaked tissue fluid from the blood vascular system and returning it to the venous circulation. They also absorb proteins, lipids, and immune cells within interstitial fluid (i.e., lymphatic fluid), transferring them to the circulation through lymph nodes. Consequently, lymphatics play a crucial role in immune surveillance throughout the body, facilitating the removal of pathogens and waste materials, as well as clearing immunoreactive components, including immune cells, from local tissues.

**Figure 1:**
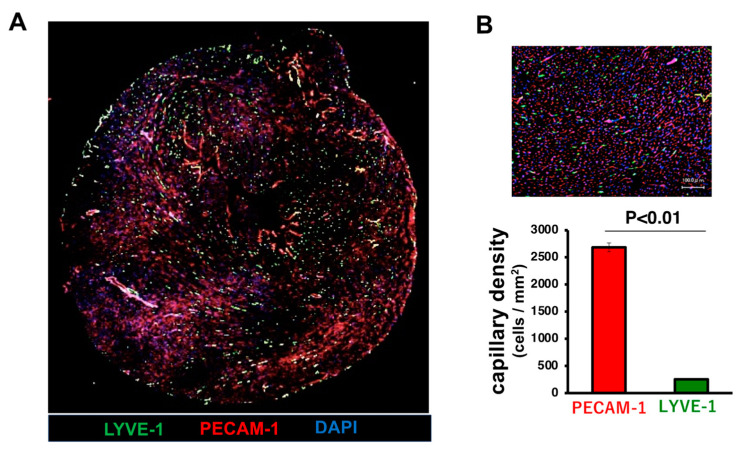
Cardiac lymphatic vessels in a normal mouse heart. (**A**) Representative picture of LYVE1 (green), CD31 (red), and DAPI (blue). It is observed that lymphatic capillaries in the heart are uniformly distributed throughout the left ventricle. (**B**) Quantitative analysis of immunostaining with LYVE-1 and CD31, respectively. Scale bar = 100 μm. Data are mean ± SEM, *p* < 0.01 by unpaired Student’s *t*-test. The lymphatic capillary density was observed to be approximately one-tenth that of the vascular density.

Although tissue lymphatic vessels also exist in the heart (Figure 1), their physiological function is little understood. Furthermore, their involvement and severity in various pathological hearts remain unclear. A recent explosion of remarkable research in this field has begun to put cardiac lymphatics in the spotlight. Accordingly, this review article will summarize reports regarding the role of the cardiac lymphatic vessels, especially in terms of various heart diseases from animal models to clinical status.

## 2. Animal Experimental Study

### 2.1. Normal Heart and Cardiac Lymphatic Vessels

#### 2.1.1. Anatomy of Lymphatic Vessels in the Heart

Initial reports on the lymphatic vessels of the mammalian heart discussed the distribution and ultrastructure of the cardiac lymphatic vessels in relation to their role in removing interstitial fluid from the heart [1,2]. A plexus of thin-walled lymphatic vessels was reported to extend throughout the epicardial, myocardial, and subendocardial regions. In the study, differences in lymphatic capillary microstructure by localization within the myocardium were also revealed. Quantitative morphologic changes were attributed to physiological (i.e., myocardial movement and metabolic activity) and morphological differences (i.e., thickness of the connective tissue surrounding the lymphatic capillaries) among the three cardiac layers [2]. These findings will provide an important basis for further research on the lymphatic vessel’s role in the physiological function of the heart.

#### 2.1.2. Discoveries of the Markers for Lymphatic Endothelial Cells

Since the reports of lymphatic artery hyaluronan receptor-1 (LYVE-1) [3,4], membrane glycoprotein podoplanin [5], vascular endothelial growth factor receptor-3 (VEGFR-3) [6], Prospero-related homeodomain transcription factor (Prox1) [7], etc. as lymphatic vessel-specific markers around the 2000s, immunostaining methods for these markers have been frequently used to identify lymphatic vessels in vivo (Figure 1). These methods have made it possible to easily visualize and evaluate the number, distribution, and morphology of lymphatic vessels, and have accelerated the pace of lymphatic vessel research.

#### 2.1.3. Origin of Cardiac Lymphatics

The origin of cardiac lymphatics remains controversial. Sabin et al. proposed a venous origin model for lymphatic vessel development (https://doi.org/10.1002/aja.1000010310). The authors intended to examine the origin and development of the lymphatic system, specifically highlighting the formation of the cardiac lymphatic and thoracic ducts in pigs. Through injection experiments and anatomical observations, they revealed the fact that lymphatic vessels develop from veins. On the other hand, Klotz et al. showed that the lymphatic vasculature of the embryonic mouse heart was composed of a heterogeneous cell population derived from both epicardial venous endothelium and lymphatic endothelial progenitor cells originating from yolk sac hematogenous endothelium [8].

#### 2.1.4. Impact of Cardiac Lymphatic Vessel Function on a Healthy Heart

A dog study was performed to elucidate the impact of acute cardiac lymphatic obstruction on left ventricular (LV) systolic and/or diastolic function [9]. It demonstrated that cardiac lymphatic obstruction impaired contractility and active relaxation, and induced mild left ventricular myocardial edema, but did not affect diastolic stiffness in the acute phase [9]. More recently, we established a murine cardiac lymphatic insufficiency model by ablating the cardiac lymphatic collector vessels and observed the impact on cardiac function both in physiological and pathologic settings in the chronic phase [10]. We demonstrated that cardiac lymphatic insufficiency itself contributes to the development of myocardial edema formation, inflammation, and fibrosis, which lead to cardiac hypertrophy, resulting in cardiac diastolic dysfunction [10]. In addition, we demonstrated that therapeutic lymphangiogenesis aimed at reconstructing the cardiac lymphatic network could rescue those adverse morphologic changes and heart failure caused by cardiac lymphatic insufficiencies [10].

Lymphangiogenesis in the heart is also associated with exercise-induced cardiac growth. Using mouse models and in vitro cellular experiments, Bei et al. found that exercise promoted cardiac lymphangiogenesis and that lymphangiogenesis was strongly associated with cardiomyocyte proliferation and hypertrophy [11]. They showed that extracellular factors released from lymphatic endothelial cells (LEC) promote proliferation and hypertrophy of cardiomyocytes [11].

### 2.2. Heart Disease Model

#### 2.2.1. Myocardial Infarction Model

The function of cardiac lymphatic vessels and their importance in the process of inflammatory regression after acute myocardial infarction (MI) were widely reported in experimental studies (Table 1). The expressions of lymphatic endothelium-specific growth factor, vascular endothelial growth factor (VEGF)-C, and its receptor VEGFR-3 were upregulated in a time-dependent manner in the acute phase just after myocardial ischemic injury [12]. In addition, proliferative lymphatic endothelial cells in damaged myocardium were observed from day 3 after injury [12]. The study demonstrated that pharmacological inhibition of the endogenous lymphangiogenic response with MAZ51 exacerbated ischemia-induced heart failure [12]. Furthermore, genetic inhibition of lymphangiogenesis also clearly resulted in higher mortality after MI in the transgenic mouse model, which expressed a soluble decoy VEGFR3 under the K14 promoter [13].

**Figure 2:**
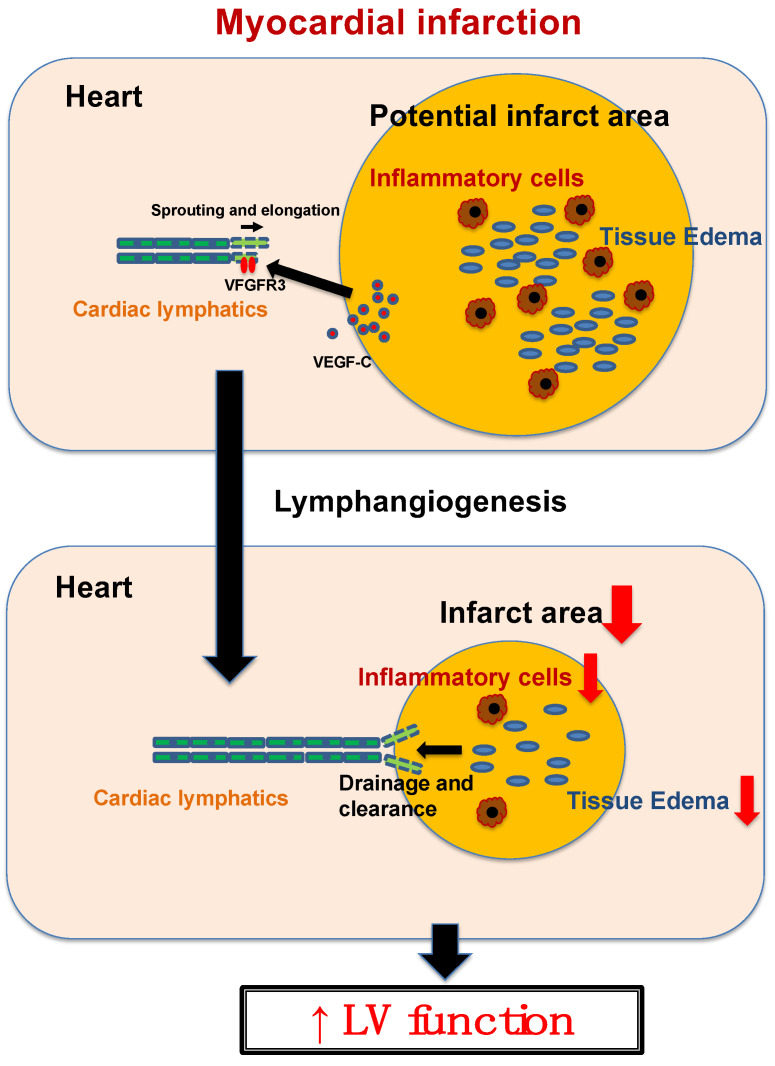
Mechanistic schema of the role of cardiac lymphangiogenesis against myocardial infarction.

On this basis, the application of VEGF-C to the MI therapy emerged (Figure 2) [8,14,15,16]. For example, the delivery of VEGF-CC156S via adeno-associated viral vectors significantly enhanced cardiac lymphangiogenesis, resulting in decreased cardiac inflammation and improved function by three weeks post-myocardial infarction [16]. At the same time, other strategies had also been conducted for the MI therapy, enhancing lymphangiogenesis while targeting another pathway. For instance, endovascular intramyocardial AdVEGF-D^ΔNΔC^ gene therapy proved to be safe and effective in the porcine acute myocardial infarction model [17]. Adrenomedullin drove reparative cardiac lymphangiogenesis, improving myocardial edema after MI [18]. Apelin in the ischemic heart was reported to restore functional lymphatic vasculature, reduce matrix remodeling, and provide protection against inflammation [19]. Lymphatic endothelial S1pr1 augmented lymphangiogenesis to resolve excessive inflammation and ameliorate adverse cardiac remodeling following myocardial infarction [20]. In the context of cell therapy, Iwamiya et al. found that VCAM1-expressing cardiac fibroblasts (CFs) could strongly promote cardiac lymphatic vessels and improve heart function [21]. Importantly, many reports have shown that promoting cardiac lymphangiogenesis led to cardioprotective effects after MI and contributed to the suppression of heart failure.

#### 2.2.2. Myocardial Ischemia-Reperfusion Injury Model

The protective role of cardiac lymphangiogenesis in cardiac remodeling after myocardial ischemia/reperfusion (I/R) injury has also been examined in multiple studies (Table 1) [12,22,23,24,25,26]. For instance, we established an I/R model and found that the expression of VEGF-C and its receptor increased, leading to increased lymphatic vessel density [12]. Interestingly, inhibiting this response exacerbated inflammation and cardiac dysfunction, while enhancing these reactions had a beneficial effect against myocardial I/R injury [12]. Moreover, Glinton et al. observed that defective efferocytosis by macrophages led to reduced cardiac lymphangiogenesis and vegfc expression after myocardial infarction. Macrophage-derived VEGF-C promoted healing through myocardial lymphangiogenesis and the suppression of inflammatory cytokines [27].

**Table 1:** Pathological change of Myocardial infarction model and ischemia-reperfusion injury model.

	Study	Year	Pathological Change of Mouse Model	Proposed Mechanisms
MI model	Klotz L. et al. [8]	2015	Lymphangiogenesis promote cardiac repair following injury	VEGF-C(C156S) treated
	Shimizu Y. et al. [12]	2018	Inhibition of lymphangiogenesis exacerbates ischemic-induced heart failure	VEGFR-3 inhibitor MAZ51, VEGF-C neutralizing antibody
	Vuorio T. et al. [13]	2018	Inhibition of lymphangiogenesis presented higher mortality after MI	Downregulation of VEGFR3 signaling modifies the structure of cardiac lymphatic network
	Henri o. et al. [14]	2016	Lymphangiogenesis improve myocardial fluid balance, cardiac remodeling and dysfunction	VEGF-C(C152S) treated
	Vieira JM. et al. [15]	2018	Lymphangiogenesis improves clearance of the acute inflammatory response	VEGF-C(C156S) treated
	Houssari M. et al. [16]	2020	Lymphangiogenesis reduced cardiac inflammation and dysfunction	VEGF-C(C156S) treated
	Pajula J. et al. [17]	2022	Lymphangiogenesis the amount of pericardial fluid and level of vascular permeability had returned to normal by day 21	Adenoviral VEGF-D (DeltaN DeltaC) gene therapy
	Trincot CE. et al. [18]	2019	Lymphangiogenesis and cardiac function post-myocardial infarction while suppressing cardiac edema	Adrenomedullin induce cardiac lymphangiogenesis via Connexin 43
	Tatin F. et al. [19]	2017	Restore functional lymphatic vasculature and to reduce matrix remodeling and inflammation	Apelin plays a key role in lymphatic vessel maturation and stability in pathological settings
	Li Q. et al. [20]	2022	Resolve excessive inflammation and to ameliorate adverse cardiac remodeling	S1pr1 tightly controlled LEC functions and homeostasis
	Iwamiya T. et al. [21]	2020	Restore the mechanical properties of ventricular walls	VCAM1-expressing cardiac fibroblasts (CFs) treated
IR model	Michael. LH. et al. [23]	1985	Release functionally active glycogen phosphorylase and CK	Reflect changes in myocardial cell egress of macromolecules and/or cell death
	Yotsumoto G. et al. [24]	1998	Alleviates the myocardial edema formation	Active drainage of cardiac lymph by hyaluronidase
	Santos AC. et al. [25]	1998	Preventing cardiac edema	Cardiac lymphatics improved with time
	Dreyer WJ. et al. [22]	1989	Chemotactic activity is generated in ischemic and reperfused myocardium	Activate the following proinflammatory functions in neutrophils, CD11b/CD18 levels were greater than neutrophils obtained before or during occlusion
	Shimizu Y. et al. [12]	2018	Inhibition of lymphangiogenesis exacerbates ischemia-induced heart failure	VEGFR-3 inhibitor MAZ51, VEGF-C neutralizing antibody
	Wang C. et al. [26]	2022	Reduce myocardial fibrosis and improve heart function	M2b macrophages can promote lymphangiogenesis
	Glinton KE. et al. [27]	2022	Macrophages promote healing	Macrophages promote myocardial lymphangiogenesis and suppress inflammatory cytokines

#### 2.2.3. Heart Transplantation Model

The cardiac lymphatic system plays a role in maintaining interstitial fluid homeostasis, regulating inflammatory responses, and modulating immune reaction in animal studies of the heart transplantation model. Although the impact of cardiac lymphangiogenesis on the allograft heart survival rate remains controversial, chemokines, cytokines, and growth factors would connect lymphangiogenesis with innate adaptive immunity during cardiac transplantation [28]. Dashkevich et al. demonstrated that pharmacological activation of the VEGF-C/VEGFR3 pathway could regulate the activity of lymphatic vessels, thereby affecting the inflammatory response and rejection of grafts. In contrast, pharmacological inhibition of VEGF-C/VEGFR3 during cardiac allograft IR injury attenuated early lymphangiogenesis, resulting in acute and chronic graft rejection [29]. The authors suggested that inhibiting the VEGF-C/VEGFR3 pathway during ischemia-reperfusion injury in solid organ transplantation could serve as a lymphatic-targeted immunomodulatory therapy to prevent both acute and chronic rejection [29]. Regarding atherosclerosis after transplantation, Chen et al. reported targeting lymphangiogenesis could be a new approach to preventing graft atherosclerosis. It was also demonstrated that early inhibition of lymphangiogenesis could alleviate graft atherosclerosis over a long time, possibly due to adaptive changes in allograft vessels disease, suggesting its potential target for the immunosuppression of graft vascular disease [30].

#### 2.2.4. Transverse Aortic Constriction Model

Transverse aortic constriction (TAC) is a surgical procedure used in cardiac research to induce cardiac pressure overload. It involves constricting the transverse aorta, resulting in an acute increase in cardiac stress and workload. TAC is usually performed in mice to study the effects of pressure overload on cardiac function and the development of heart failure [31]. Heron et al. demonstrated endogenous lymphangiogenesis-limited cardiac inflammation and perivascular fibrosis, which delayed the development of heart failure in C57Bl/6 mice but not in Balb/c mice [32]. Moreover, Lin et al. reported that activation of the VEGF-C/VEGFR-3 axis exerts a protective effect during the progression from cardiac hypertrophy to heart failure, emphasizing selective stimulation of cardiac lymphangiogenesis as a promising therapeutic strategy for hypertrophic heart diseases [33]. Interestingly, another study contrary showed that LCZ696, a combination monodrug of angiotensin type 1 receptors and neprilysin inhibitors, suppressed the inflammatory response, ameliorating cardiac hypertrophy, fibrosis, and the cardiac lymphatic remodeling (i.e., upregulation of VEGF-C, VEGFR3, and LYVE-1 expression) regardless of the cause or effect causality in a mouse model of TAC-induced cardiac hypertrophy [34].

Bizou et al. observed a remodeling of the lymphatic network and a decrease in lymphatic efficiency following TAC-induced heart failure [35]. Of note, they highlighted that specific macrophage subpopulations regulated cardiac lymphatics during pathological hypertrophy in a TAC model and might constitute a key mechanism underlying the progression of heart failure [35].

#### 2.2.5. Angiotensin II–Induced Hypertension and Cardiac Hypertrophy Model

Past studies suggested that angiotensin II (Ang II) would induce cardiac edema and hypertrophic remodeling by a lymphatics-dependent mechanism. Using lymphatics-specific VEGFR-3 knockout mice and epoxomicin interventions, Bai et al. found that both lymphangiogenesis and lymphatic barrier hyperpermeability in the heart were involved in adaptive hypertrophic remodeling and dysfunction induced by Ang II [36]. Additionally, SIRT3 was found to be a positive regulator of lymphangiogenesis, which plays a protective role in hypertensive heart injury [37]. Moreover, Song et al. emphasized that treatment with VEGFC_C156S_ improved cardiac lymphatic function, alleviated fibrosis and inflammation, and reduced hypertension, thereby preventing cardiac dysfunction in an angiotensin II-induced heart failure [38]. They advocated the specific modulation of angiotensin II-induced inflammatory responses in the heart by therapeutic lymphangiogenesis [38].

#### 2.2.6. Myocarditis Model

Although lymphatic systems play a key role in a lot of inflammatory diseases, the role of lymphatic vessels in myocarditis is largely unknown. Omura et al. showed the relationship between myocarditis caused by Theiler virus infection and cardiac lymphatics [39]. They proposed that Theiler’s murine encephalomyelitis virus (TMEV) infection results in dysfunction of cardiac lymphatics, attributed to pro-inflammatory cytokines triggered by the inflammatory response [39]. This dysfunction may reduce the contractility of the cardiac lymphatic muscle, thereby impairing lymphatic fluid flow [39]. Such a mechanism could be a significant factor in the progression of myocarditis [31]. Additionally, the same group demonstrated that IL-1β diminishes contractility in rat cardiac lymphatic muscle cells through the COX-2/PGE2 signaling pathway, with synergistic involvement of TNF-α. These pathways were proposed to facilitate the accumulation of inflammatory mediators within the heart, contributing to the progression from acute myocarditis to dilated cardiomyopathy [40].

#### 2.2.7. Arrhythmia Model

Some articles have suggested the role of cardiac lymphatics in pathologic lesions involving the conduction system. Uhley et al. discussed lymphatics potential role in pathologic injuries involving the conduction system, either directly or indirectly, by carrying large amounts of potassium in high concentrations from distantly damaged areas [41]. The effects of potassium manifest in the form of conduction defects due to decreased conduction velocity or in the development of cardiac arrhythmias [41].

An interesting study created artificial lymphatic cardiomyopathy in 30 dogs by ligating regional lymph nodes and efferent lymphatic collecting stems from the heart. During the development of myocardial necrosis, repeated electrocardiographic recordings showed the development of arrhythmias and conduction disturbances [42]. In the “lymphatic cardiomyopathy,” arrhythmias were exacerbated, and the pacing function of the sinus node deteriorated [42]. Cardiac lymphatic insufficiency might be associated with the pathogenesis of certain arrhythmias [42].

#### 2.2.8. Valvular Disease Model

Recent research has identified a novel mechanism of inflammation-associated, VEGFR3-dependent postnatal lymphangiogenesis in a murine model of autoimmune valvular carditis, mimicking human rheumatic heart disease [43]. The author demonstrated that inhibition of VEGFR3 through various approaches effectively suppressed the expansion of the mitral valve lymphatic network [43]. Echocardiographic analysis revealed that the treated mice exhibited left ventricular dysfunction and mitral valve stenosis [43]. Furthermore, valve lymphatic density was observed to increase progressively with age in their mice; a trend that correlated with the exacerbation of ventricular dysfunction [43].

## 3. Clinical Evidence

Both practicing cardiologists and cardiac surgeons have devoted their energies to targeting the heart’s blood vessels, while no consideration has been given to the cardiac lymphatic vessels. This is because lymphatic research has lagged behind vascular research, and evidence regarding the role and importance of the cardiac lymphatics has long been lacking. The identification and visualization of the microstructure and intracardiac distribution of cardiac lymphatic vessels became possible after the first lymphatic-specific markers (i.e., lymphatic artery hyaluronan receptor-1 [LYVE-1] [3,4], membrane glycoprotein podoplanin [5], vascular endothelial growth factor receptor-3 [VEGFR-3] [6], and Prospero-related homeodomain transcription factor [Prox1] [7]) were reported around the year 2000. Since then, the development of technology to knock out lymphatics-related genes has made it possible to analyze the functional role of lymphatic vessels in mice, and lymphatic vessel research has gradually progressed, and its importance has begun to be reevaluated with the accumulation of knowledge. Furthermore, the possibility of therapeutic lymphangiogenesis in various pathological conditions using small animals has been reported one after another in the past few years. Therefore, it is highly expected that clinical studies for intractable cardiac diseases will be developed and conducted within the next few years.

### 3.1. Ischemic Heart Disease

Along with new pathological findings in terminal heart failure including ischemic cardiomyopathy, chronic myocardial edema was reported to be a common phenomenon [44]. Myocardial edema would induce further deterioration of systolic and diastolic function, which has been associated with the development of interstitial myocardial fibrosis [45]. However, the alteration of cardiac lymphatic microvasculature in terminal heart failure is poorly understood. Dashkevich et al. reported that although the absolute densities of all markers (i.e., LYVE-1, PROX-1, and VEGFR-3) did not differ significantly between failing and normal hearts, the proportion of open LYVE-1-positive lymphatics was significantly higher in failing hearts than in controls [46]. The result indicated that in the context of end-stage heart failure, the proliferation of primary lymphatic vessels predominantly occurs through coordinated growth mechanisms, as opposed to the “de novo” generation from stem or progenitor cells, or through sprouting from pre-existing venous vessels [46]. Another study demonstrated that cardiac lymphangiogenesis lagged behind blood vessel angiogenesis in myocardial remodeling after MI [47]. Therefore, the findings suggest that newly formed lymphatics may play a primary role in the maturation of fibrosis and scar formation by facilitating the drainage of excess proteins and fluid after MI [47].

### 3.2. Cardiomyopathy

Cardiac lymphatic dysfunction occurrence in patients with hypertrophic obstructive cardiomyopathy (HOCM) or dilated cardiomyopathy (DCM), as well as the associations between lymphatic vessels with myocardial cardiac fibrosis have been investigated. Zhou et al. exhibited the LYVE1 expression levels by bioinformatics analysis, suggesting that the gene might be involved in the development of DCM-induced HF [48]. Moreover, Jiang et al. demonstrated that LYVE-1 positive lymphatics have a close association with ventricular septal fibrosis in HOCM patients [49]. In particular, they found patients with syncope symptoms had higher levels of lymphatic micro-vessel density [49]. Dashkevich et al. discovered that DCM hearts showed a significantly higher density of LYVE-1 positive lymphatics, whereas no difference was seen for other markers (i.e., VEGFR-3, D2-40, and PROX-1) compared to normal donor hearts [50]. In contrast, ICM hearts showed a significantly higher density of D2-40 positive lymphatics and a lower density of VEGFR-2 capillaries [50]. These findings indicated that the effect of microvascular remodeling may be substantially different between two clinically important causes of cardiomyopathy [50]. Additionally, another study reported lymphatic vessel marker D2-40 might help to distinguish clinical cardiac sarcoidosis without granuloma from DCM [51].

### 3.3. Myocarditis

Reports on the presence of myocarditis and cardiac lymphatic vessels in clinical samples are very limited. An increase in the number of cardiac lymphatic vessels has been observed in myocarditis [52]. Lymphatics accompanied inflammatory cells in the myocardial interstitium [52]. On the other hand, blood vessels did not increase in myocarditis in the study [52].

### 3.4. Valvular Disease

Calcific aortic stenosis (CAS), the most common valvular disease in developed countries currently, is categorized into two main types: senile and congenital malformations. Studies have shown that the pathogenesis of CAS is related to inflammation, with the presence of both vascular and lymphatic vessels in calcified aortic valve leaflets, associated with lymphocytic infiltration [53]. These findings further confirm the importance of inflammation in the pathophysiology of CAS and the potential link to cardiovascular lymphatic vessels [53].

### 3.5. Arrhythmia

Postoperative atrial fibrillation (AF) has been associated with several risk factors, including inadequate protection of the right atrial tissue during aortic cross-clamping and histopathological alterations in the right atrium. For instance, coronary artery bypass grafting (CABG) necessitates the removal of adventitial tissue and the aortic fat pad from the ascending aorta, both of which house the sinoatrial (SA) node lymphatic collector. Damage to the SA node lymphatic collector can impair lymphatic drainage from the SA node, subsequently leading to dysfunction of the cardiac conduction system, such as sick sinus syndrome. This disruption contributes to the development of postoperative AF [54]. Moreover, dilated lymphatic channels were observed in regions of dense, mature fibrosis in patients with hypertrophic cardiomyopathy (HCM) [55]. These complex channels may promote pro-arrhythmic conduction, a previously recognized substrate for malignant ventricular arrhythmia. Importantly, this finding suggested a new imaging marker for HCM patients at high risk of malignant arrhythmias [55].

### 3.6. Heart Failure

In chronic heart failure (CHF), a significant increase in thoracic duct (TD) flow is generally observed [56], and two major factors influence this flow. One factor is elevated venous pressures that generate an excessively high flux of water into the interstitial space. The other factor is the removal of interstitial fluid and its return to the venous system. Flow through the TD is impeded by elevated central venous pressure (CVP). Moreover, increased interstitial fluid (i.e., myocardial edema) interferes with systolic and diastolic myocardial function, contributing to worsening heart failure as CVP rises [57]. In this regard, Fudim et al. suggested that by addressing dysregulation of the lymphatic system, it is possible to alleviate symptoms and improve prognosis in patients with heart failure [58]. Itkin et al. demonstrated the feasibility of pharmacological modulation of the lymphatic system and intervention methods to improve lymphatic circulation and tissue congestion in heart failure patients [59]. Most hospitalizations and symptoms related to heart failure are primarily driven by manifestations of venous congestion rather than reduced cardiac output. Several mechanisms contributing to interstitial fluid accumulation, involving the lymphatic system, have been previously described [58]. Panagides et al. showed that transcatheter percutaneous lymphatic drainage has emerged as a feasible and promising technique for alleviating congestion and interstitial edema in heart failure patients [60]. Additionally, in acute heart failure (AHF), a low level of VEGF-C was reported to associate with more severe signs of congestion, such as lower-extremity edema and ascites. Thus, VEGF-C supplementation would play a role in lymphatic system development, which may serve as a mechanistic link to the severity of symptoms and outcomes in AHF [61].

### 3.7. Heart Transplantation

Development of cardiac allograft vasculopathy (CAV), is the main limitation of long-term survival of heart transplant patients [62]. CAV was hypothesized to be a result of interrupted lymphatic drainage after surgery [63]. Edwards et al. used SPECT/CT lymphoscintigraphy to provide objective quantification of lymphatic flow in a pre-clinical model of chronic heart transplant rejection and correlated it with graft damage and inflammatory infiltrates to investigate the link between functional lymphatic flow and the alloimmune process. They found that chronic rejection led to increased lymphatic flow from the donor graft to the draining lymph nodes, which may contribute to enhanced cellular trafficking, alloimmunity, and cardiac allograft vasculopathy [64]. Moreover, Subendocardial lymphonodular aggregates, known as Quilty lesions, are frequently observed (in up to 40%) in human cardiac allografts [65]. Yamani et al. revealed the inflammatory compartment of Quilty lesions was of recipient origin and showed chimeric neoangiogenesis of blood and lymphatic vessels [66]. Furthermore, another study demonstrated that the appearance of Quilty lesions results in improved long-term graft survival [67].

### 3.8. Congenital Diseases

Congenital heart disease refers to structural abnormalities in the heart, or large blood vessels of the heart, during fetal development, resulting in impaired heart function. Congenital heart disease can be divided into multiple types based on different pathophysiological mechanisms, including single ventricle disease, ventricular septal defect, and patent ductus arteriosus. Individuals with congenital heart disease often face a series of complex physiological problems due to structural abnormalities of the heart. Studies have shown that congenital heart disease is closely related to various lymphatic flow disorders, such as chylothorax, plastic bronchitis, and protein-losing enteropathy [68,69]. In patients with congenital heart disease, the lymphatic system is often affected, especially in individuals with single-ventricle heart disease [70]. Abnormal structure of the heart may lead to poor development of lymphatic vessels, thereby affecting lymphatic drainage. The normal development of lymphatic vessels is essential for maintaining fluid balance and immune function in the body.

## 4. Limitation and Future Perspective

Cardiac surgeons and thoracic surgeons have not emphasized the care and preservation of cardiac lymphatic anatomy in surgery, including heart transplantation. Also, for example, the reconstruction of cardiac lymphatic vessels for transplanted hearts has not been performed at all. However, a review of the past literature summarized in this article suggests that the cardiac lymphatic vessels play an important role in maintaining cardiac homeostasis and that targeting the lymphatic vessels or adding lymphatic reconstruction therapy in conjunction with conventional therapy may help prevent disease progression and improve treatment outcomes.

## 5. Conclusions

Although clinical studies on cardiac lymphatics are still limited, progress is being made in elucidating various pathological conditions through animal models. As summarized in this review, cardiac lymphatics are closely linked to the pathogenesis and progression of heart diseases, in conjunction with the dynamics of inflammation, edema, fibrosis, and other factors within the heart. Further corroboration of experimental evidence in clinical samples is needed. In addition, therapeutic applications targeting the cardiac lymphatic systems are expected to be applied to heart disease in actual clinical practice in the future.

## Data Availability

The data are available from the corresponding author upon reasonable request.
